# Chip Formation Mechanisms When Cutting Amorphous Alloy with Cubic Boron Nitride Tools Based on Constitutive Equation Parameter Optimisation

**DOI:** 10.3390/mi16050534

**Published:** 2025-04-29

**Authors:** Jinguang Du, Dingkun Wang, Yaoxuan Guo, Wuyi Ming, Wenbin He

**Affiliations:** 1 Henan Provincial Key Laboratory of Intelligent Manufacturing of Mechanical Equipment, Zhengzhou University of Light Industry, Zhengzhou 450002, China; dujinguang@zzuli.edu.cn (J.D.); wdingkun0707@163.com (D.W.); guo15729117707@163.com (Y.G.); mingwuyi@gmail.com (W.M.); 2 Henan International Joint Laboratory of Complex Mechanical Equipment Intelligent Monitoring and Control, Zhengzhou 450002, China; 3 Guangdong Provincial Key Laboratory of Digital Manufacturing Equipment and Guangdong HUST Industrial Technology Research Institute, Huazhong University of Science and Technology, Dongguan 523808, China

**Keywords:** amorphous alloy, JC constitutive equation, finite element models, chip formation mechanism

## Abstract

Owing to potential inaccuracies in the current stress–strain curve used for constructing the Johnson–Cook (JC) constitutive model of amorphous alloys, the parameters of the JC constitutive equation were derived using Oxley’s cutting theory, negative chamfer theoretical mechanics modelling, and the particle swarm optimisation algorithm. A two-dimensional finite element cutting model was subsequently established using AdvantEdge software. The optimised constitutive model was used to simulate the main cutting force (Fz) and the backward force (Fy), which resulted in average errors of 12.461% and 9.161%, respectively. Based on the optimised constitutive model in which the JC constitutive equation parameters were derived using Oxley’s method, the variations in temperature, strain rate, and stress in the deformation zone during the cutting process were analysed. The chip microstructures revealed the transformation of lamellar chips into serrated chips resulting from a combination of plastic deformation, adiabatic shear, and shear slip.

## 1. Introduction

Amorphous alloys, also called metallic glasses, are novel high-performance alloys characterised by long-range disordered and short-range ordered amorphous structures, which are produced by rapidly cooling and crystallising a high-temperature liquid melt. The atomically disordered structure imparts exceptional compressive strength, hardness [1], fracture toughness, elastic limit [2], wear resistance [3], and magnetic properties [4] to amorphous alloys. These alloys have been widely applied in cutting-edge fields such as aerospace, electronic devices, medical machinery, and sports equipment [5,6]. To fully realise their potential, machining amorphous alloys into machine parts is essential. The technology used for machining and manufacturing amorphous alloys plays a crucial role in determining their engineering applications.

In recent years, many scholars have investigated the chip morphology of amorphous alloys through cutting experiments, leading to the discovery of numerous significant machining mechanisms. Bakkal et al. [7] found that when cutting Zr_41.2_Ti_13.8_Cu_12.5_Ni_10_Be_22.5_ (Vit1), the chip morphology with a fire emission phenomenon exhibited a melted state. Fujita et al. [8] obtained uniformly spaced continuous lamellar chips during the turning of Zr_65_Cu_15_Ni_10_Al_10_ and Pd_40_Cu_30_Ni_10_P_20_. The slip mechanisms responsible for this chip morphology were controlled by the shear stress on the shear surface. Jiang et al. [9] furthered the research conducted by Fujita [8] by quantitatively characterising the mechanism behind the formation of lamellar chips while cutting Vit1. Dhale et al. [10] analysed the serrated chip morphology obtained during the orthogonal cutting of Zr_67_Cu_10.6_Ni_9.8_Ti_8.8_Be_3.8_ (Vit1b-X). They found that increasing the cutting speed led to severe material tearing, with slight shear bands on individual serrated chip units extending inward, accompanied by crushing cracks, material fractures, and larger serration spacing. The arrangement of the atomic amorphous structure results in a free volume inside the amorphous alloy, and shear band formation is a thermodynamic coupling process dominated by stress-driven local free volume softening. Dhale et al. [11] utilised nanoindentation techniques to quantify the free volume in the subsurface shear transformation zone of Vit1b-X during machining, thereby revealing the mechanism of material softening. Ding et al. [12] suggested that the serrated chips produced by the orthogonal cutting of Vit1 were formed by cracks at the root of the non-free surface and at the free surface along the primary shear zone extension. Energy consumption during crack extension formation was also calculated from an energy perspective, and the relationship between energy and chip morphology was analysed. Ding et al. [13] observed the non-free surface morphology of chips during the high-speed cutting of Vit1. Under the action of squeezing and shearing, the outflow of chips exhibited a significant velocity gradient on the rake face of the tool owing to chip adhesion, which led to the ductile tearing and flaking of the chip material, generating many pits and residual chips on the non-free surface.

Chen et al. [14] investigated the cutting performance of Zr_57_Cu_20_Al_10_Ni_8_Ti_5_ (Zr57BMG) and Zr_48_Cu_47.5_Co_0.5_A_l4_ (Zr48BMGC) at low speeds and found that the normal force was greater than the main cutting force for both materials. This is due to the low modulus of elasticity of both materials, which causes them to bend and deform in the radial direction during cutting. Although Zr57BMG has a higher hardness and yield strength than Zr48BMGC, its cutting force is lower than that of Zr48BMGC. This is primarily because in the shear deformation zone, the frictional contact between the tool and material generates heat that cannot be dissipated promptly. Yang et al. [15] also concluded that, owing to the low thermal conductivity of Zr57BMG, the thermal softening effect during the machining process reduces the main cutting forces. Although the hardness and yield strength of Zr57BMG are significantly higher than those of Zr702, the difference in the main cutting forces between these two materials is insignificant. Deng et al. [16] performed a fast Fourier transform (FFT) on the main cutting force and cutting temperature at constant speed during the machining of Vit1. The cutting force and temperature exhibited periodic changes consistent with the serrated chip formation pattern. Increased temperatures, tool wear, and chip adhesion after tool breakage can significantly impact the cutting force. Wang et al. [17,18] investigated the micro-milling process of Vit1 and found that the cutting force decreased linearly as the feed rate was reduced. However, when the feed per tooth was less than 1 μm/z, a non-linear phenomenon occurred in the cutting force, primarily caused by the minimum undeformed chip thickness, and the main material removal mode was ploughing [17,18].

Using finite element models (FEMs) to investigate the cutting mechanism of an amorphous alloy allows for an intuitive understanding of the chip formation process and significantly reduces machining costs. Owing to the amorphous structure of these materials, developing a constitutive model of the materials is complex. Numerous scholars have conducted extensive research to solve the parameters of the constitutive equation and compare the accuracy of the model with chip morphology and cutting force. Shen et al. [19] considered the influence of hydrostatic stress and developed a new D-P constitutive model. Through comparison with experiments, the high credibility of this model was verified. He et al. [20] established a new adiabatic constitutive model and conducted two-dimensional cutting simulations and micro-cutting experiments on Fe_40_Ni_40_P_14_B_6_. They found that during the cutting process, temperature fluctuations and periodic stress changes within the shear bands were the primary causes of sawtooth-shaped chip formation [20]. The simulation results showed strong agreement with the experimental findings. Taking into account the effect of the intermediate principal stress on the yield, Jin et al. [21] derived the Drucker–Prager (D-P) constitutive model by converting the parameter equation of the M-C constitutive model of Vit1. Zhang et al. [22] divided the D-P constitutive model equation based on the thermodynamic properties of Vit1 using the Vit1 transition temperature (*T*_g_) as the segmentation point. Based on data from compression, tension, and cutting experiments, the material parameters in the model were derived considering the effect of hydrostatic stress. A D-P constitutive model was then established to better match the actual Vit1 material properties.

Wang et al. [23] suggested that the Johnson–Cook (JC) constitutive equations originate from the material stress state and elastic energy accumulation/release processes and have no relationship with the structure of crystalline or amorphous phases. Based on the results of extensive tensile and compressive experiments, a JC constitutive model was constructed to describe the flow stress of metallic glasses. Park et al. [24] believed that the constitutive model of metallic glasses was designed to describe their mechanical behaviours in the supercooled liquid region rather than at room temperature. Because of the low thermal conductivity and high strain rate of metallic glasses, the heat generated by shear deformation cannot be effectively transferred. When performing sapphire laser-assisted cutting of Zr_52.5_Ti_5_Cu_17.9_Ni_14.6_Al_10_ (Vit1-105), assuming that the cutting temperature is lower than *T*_g_, Vit1-105 can be described using a JC constitutive model. Chau et al. [25] fitted the JC constitutive model parameters of Zr_55_Cu_30_Al_10_Ni_5_ through split Hopkinson pressure bar experiments and analysed the periodic fluctuations of the cutting force in simulations and experiments, which effectively explained the formation process of serrated chips.

In summary, the current research on chip formation and cutting forces while cutting amorphous alloys primarily involves cutting experiments supplemented by finite element simulations to investigate the material removal mechanism. However, studies on the parametric solution of the JC constitutive expression for amorphous alloys in FEM cutting operations are limited. Additionally, there is little research on using orthogonal experiments and mechanical theory modelling to predict the Vit1 constitutive model. Therefore, this study established a two-dimensional cutting force prediction model that takes into account the negative chamfered structure of the tool edge. The particle swarm optimisation (PSO) algorithm was used to iteratively obtain the parameters of the JC constitutive model, and the reliability of the model was confirmed through experimental and simulated comparisons. Based on the validated simulation model and microscopic chip morphology, variation patterns in the flow stress, strain rate, and temperature in the shear deformation zone were investigated to reveal the chip formation mechanism.

## 2. Materials and Methods

### 2.1. Experiments

#### 2.1.1. Workpiece and Cutting Tool

The workpiece material was a cylindrical Vit1 bar with diameter of 10 mm and length of 150 mm, produced by Pan Xing New Metal (Changzhou, China).

Considering previous research related to Vit1, tool material toughness, and Vit1 properties, this study used cubic boron nitride (CBN) tools for cutting experiments with Vit1 amorphous alloy. The CBN tool was obtained from Hualing Superhard Company (Zhengzhou, China) with tool model TNGA160404S01025-6S and grade BN-H11. The CBN hard grain content was 70%, and the average grain size ranged from 2 to 4 μm. TiN was used as the binder phase, and no coating was applied to the tool surface. The tool tip had a radius of 0.4 mm, and the rake and flank angles were both 0°. The main cutting edge of the tool had a negative chamfer and blunt finish, resulting in a blunt cutting edge. The tool’s cutting rake and flank angles could be adjusted using a cutter bar.

#### 2.1.2. Measurements

(1) Equipment

**Figure 1:**
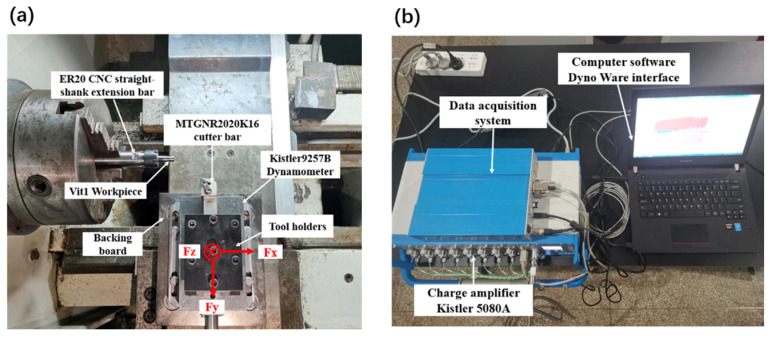
Experimental equipment. (**a**) Tool installation position; (**b**) cutting force data acquisition system.

The CNC lathe machine used in the orthogonal cutting experiments was CAK6136V/750 (Shenyang First Machine Tool Factory, Shenyang, China), which provides a speed range of 200–3000 r/min. Considering the machining characteristics of Vit1 amorphous alloy and to ensure the accuracy of the experimental data, the workpiece was reinforced and extended using an ER20 CNC straight-shank extension bar according to the Vit1 workpiece dimensions. This was performed to improve the cylindrical accuracy of the cutting machine spindle and rigidity of the workpiece, as shown in Figure 1a.

The test and measurement system for cutting force in this study consisted mainly of a force-measuring instrument (Kistler 9257 B) produced by Kistler Company (Winterthur, Switzerland), a digital signal acquisition system charge amplifier (Kistler 5080A), and supporting computer software (Dyno Ware v2.6.4.15), as illustrated in Figure 1b. The system simultaneously measured the component forces in the *X*, *Y*, and *Z* directions during the experiment. *F_z_* is the main cutting force, and its direction is consistent with the cutting speed; *F_y_* is the backward force perpendicular to the axis of the turning workpiece; and *F_x_* is the feed force, and its direction is parallel to the axis of the turning workpiece. The thickness of the chips was measured using a German Leica ultra-depth field three-dimensional microscope (DVM6) (Leica, Wetzlar, Germany), while the chip micromorphology was observed and analysed using a Phenom XL scanning electron microscope (SEM) (Thermo Fisher Scientific, Eindhoven, The Netherlands).

(2) Cutting parameters

Single-factor experiments for orthogonal cutting were designed according to the research objectives. The fixed cutting depth was 0.2 mm, and the other parameters are shown in Table 1. To avoid the effect of tool wear, a new tool was used every three turning operations with a 10 mm axial feed to ensure stable cutting force data collection. In actual operations, owing to the elastic deformation of the workpiece, a certain degree of flexural bending occurs in the overhanging part, introducing errors in the actual cutting depth. This depth corresponds to the radial component of the amount of material removed from the workpiece surface during the turning process, which is half the difference in diameter before and after turning. Therefore, theoretical calculations must consider the impact of the actual cutting depth error and substitute the actual value into the formula. As shown in Figure 1, due to the small size of the workpiece, only the left side was clamped. This approach may lead to the phenomenon of ‘tool relieving’, and during the machining process, the tool will continue to undergo wear, ultimately resulting in the actual cutting depth being less than the theoretical cutting depth (0.2 mm). According to the data in Table 1, the machining parameters have a significant impact on the actual cutting depth. When the cutting speed is 10 m/min, the actual cutting depth ranges from 0.170 to 0.185 mm; when the cutting speed was increased to 20 m/min, the actual cutting depth ranges from 0.180 to 0.185 mm.

### 2.2. Methods for Determining JC Constitutive Model Parameters 

#### 2.2.1. Fitting Stress–Strain Curve Method Parameters

The flow stress of the workpiece being machined is an important parameter for analysing the cutting process and is the foundation and prerequisite for simulating this process. The JC constitutive model provides a good description of the thermal softening, work hardening, and strain-rate strengthening effects. It reflects the degree of plastic deformation of the material during machining. The values of the five parameters *A*, *B*, *n*, *C*, and *m* of the JC model depend on the workpiece material. The specific expression of the JC constitutive model is as follows [23]:

(1) $$\sigma =\left(A+B\varepsilon^{n}\right)\left(1+C\ln\frac{\dot{\varepsilon }}{{\dot{\varepsilon }}_{0}}\right)\left[1-{\left(\frac{T-T_{r}}{T_{m}-T_{r}}\right)}^{m}\right]$$

where σ is the flow stress, *A* refers to the yield strength (MPa), *B* denotes the hardening modulus (MPa), *n* stands for the hardening factor, ε is the strain, $\dot{\varepsilon }$ stands for the strain rate (s^−1^), ${\dot{\varepsilon }}_{0}$ represents the reference strain rate (s^−1^), *C* refers to the strain reinforcement factor, *T* stands for the workpiece temperature (°C), *T_r_* denotes the reference temperature (°C), *T_m_* is the melting temperature (°C), and *m* represents the thermal softening factor.

The workpiece was produced by suction casting in a copper mould, yielding similar material parameters to the Vit1 casting method applied by Wang et al. [26] using industrial purple copper. Experimental data from the compressive mechanical property tests were used to fit the parameters *A*, *B*, and *n* of the JC constitutive equation. The Vit1 uniaxial compression test conducted by Wang et al. [27] was used to modify the strain strengthening coefficient *C* and temperature influence coefficient *m*.

(1) Determining parameters *A*, *B*, and *n*

The manufacturer specified a room temperature yield stress range of 1896–2044 MPa. According to the compressive stress–strain curves of cast specimens with different casts displayed in Ref. [25], the yield stress determined under quasi-static conditions (${\dot{\varepsilon }}_{0}$ = 5 × 10^−4^ s^−1^, *T* = 293 K) was 1964 MPa, and by interpolating data at strain values of 2.5%, 4%, and 5%, the stress values were 2012, 2070, and 2082 MPa, respectively. The last two components of the JC constitutive equation were set to 1 using the separation of variables method to eliminate the impact of the strain rate strengthening and heat softening terms on parameters *B* and *n*. The logarithm of the simplified constitutive equation can be derived as expressed in Equation (2).

(2) ln(σ−A)=ln(B)+nln(ε)

By replacing ln(σ−A) with y, substituting b for ln(B), replacing n with k, and substituting x for ln(ε), the above equation can be transformed into y=b+kx. Straight-line fitting using the Origin software (v9.6.5.169) was performed to obtain the intercept and slope, which in turn yielded *n* and *B* values of 0.7267 and 829.07 MPa, respectively.

(2) Determining parameter *C*

According to the results of stress–strain curves at different strain rates in Ref. [26], at a strain of 0.08 and a reference strain rate of 5 × 10^−4^ s^−1^, the stress values for strain rates of 2 × 10^−4^, 1 × 10^−3^, 2 × 10^−3^, and 5 × 10^−3^ s^−1^ were 146, 473, 752, and 913 MPa, respectively. Disregarding the temperature rise effect, the value of the last term of the JC constitutive equation is 1. Simplifying this equation yields Equation (3).

(3) $$\frac{\sigma }{\left(A+B\varepsilon^{n}\right)}=\left(1+C\ln\frac{\dot{\varepsilon }}{{\dot{\varepsilon }}_{0}}\right)$$

For Equation (3) above, by replacing $\frac{\sigma }{\left(A+B\varepsilon^{n}\right)}-1$ with y, substituting k for C, and replacing $\left(\ln\frac{\dot{\varepsilon }}{{\dot{\varepsilon }}_{0}}\right)$ with x, the equation y=b+kx is obtained. The intercept and slope were calculated using Origin software, and a straight-line fit produced a *C* value of 0.1171. Following the preceding methods, the interpolated values were taken at strains of 0.18 and 0.28, and *C* was calculated to be 0.1125 and 0.111, respectively, taking the average of the three calculations to be 0.1138.

(3) Determining parameter *m*

According to the results of the stress–strain curves at different temperatures in Ref. [26], at a strain of 0.08, reference strain rate of 5 × 10^−4^ s^−1^, and strain rate of 5 × 10^−3^ s^−1^, the stress values at temperatures of 633, 643, 653, 663, and 673 K were 761, 690, 440, 293, and 186 MPa, respectively. After simplifying the JC constitutive equation, Equation (4) was obtained by taking the logarithm of both sides as follows:

(4) $$\ln\left(1-\frac{\sigma }{\left(A+B\varepsilon^{n}\right)\left(1+C\ln\frac{\dot{\varepsilon }}{{\dot{\varepsilon }}_{0}}\right)}\right)=\left[m\ln\left(\frac{T-T_{r}}{T_{m}-T_{r}}\right)\right]$$

For Equation (4) above, by replacing $\ln\left(1-\frac{\sigma }{\left(A+B\varepsilon^{n}\right)\left(1+C\ln\frac{\dot{\varepsilon }}{{\dot{\varepsilon }}_{0}}\right)}\right)$ with y, substituting k for m, and replacing $\ln\left(\frac{T-T_{r}}{T_{m}-T_{r}}\right)$ with x, the equation y=b+kx can be obtained. The intercept and slope were determined by straight-line fitting using Origin software, which produced an m value of 0.0774. Following the aforementioned processes, the interpolated values were obtained at strains of 0.18 and 0.28, and *m* was then calculated to be 0.0764 and 0.0705, respectively, taking the average of the three calculations to be 0.0748.

The parameters of the JC constitutive equation for Vit1 were obtained by linear fitting, as expressed in Equation (5).

(5) $$\sigma =\left(1964+829.07\varepsilon^{0.7267}\right)\left(1+0.1138\ln\frac{\dot{\varepsilon }}{{\dot{\varepsilon }}_{0}}\right)\left[1-{\left(\frac{T-T_{r}}{T_{m}-T_{r}}\right)}^{0.0748}\right]$$

#### 2.2.2. Derivation of Parameters for Oxley’s Predictive Machining Theory

To study the cutting mechanisms of metal using finite element analysis, establishing a mechanical analysis model is essential. Most of the current research has focused on calculating shear angles to solve theoretical cutting force models, such as the shear angle calculation method proposed by Merchant [28], slip-line field theory proposed by Lee and Shaffer [28,29], and parallel surface shear region theory proposed by Oxley [30].

Based on finite element and mechanical analysis models, this study employs Oxley’s orthogonal cutting theory to consider the effects of strain, strain rate, and temperature on material flow stresses. Material deformation and temperature changes that occur throughout the cutting process have impacts on the material flow stresses and, therefore, on the cutting characteristics, such as the cutting force and chip thickness. The cutting parameters and data from the orthogonal cutting experiments were used to calculate the JC flow stress constitutive model, including shear angle, cutting speed, and cutting thickness, and to establish a cutting force prediction model that takes into account the negative chamfering structure of the tool edge. For tools with small negative chamfering widths, both the chamfered surface and rake face come into contact with the chip during cutting. The resultant cutting force (*F*_R_) refers to vector superposition of chip forming force (*R*) and the negative chamfered edge ploughing force (*F*_cf_) [31].

The resultant cutting force (*F*_R_) of the chamfered tool can also be decomposed into the cutting force *F*_C_ in the cutting direction and the cutting force *F*_T_ perpendicular to the direction of the machined surface, as expressed in Equation (6).

(6) $$\left\{\begin{array}{l} F_{R}=R+F_{\mathrm{cf}}=F_{C}+F_{T}\\ F_{C}=F_{c1}+F_{\mathrm{cf}\text{-}c};\;F_{T}=F_{t1}+F_{\mathrm{cf}\text{-}t} \end{array}\right.$$

where *F*_c1_ and *F*_t1_ denote the components of *R* parallel and perpendicular to the direction of the machined surface, respectively, while *F*_cf-c_ and *F*_cf-t_ represent the components of *F*_cf_ parallel and perpendicular to the direction of the machined surface, respectively.

(1) Mechanical analysis of shear plane AB

According to Oxley’s cutting model, assuming that the shear surface AB is located at the centre of the slip-line field in the primary deformation zone and that the strain distribution is uniform, the chip formation force can be decomposed [32].

The shear flow stress $\sigma_{AB}$ and shear angle Φ on shear plane AB were derived based on experimental data.

(7) $$\sigma_{AB}=\left(A+B{\varepsilon_{\mathrm{AB}}}^{n}\right)\left(1+C\ln\frac{{\dot{\varepsilon }}_{\mathrm{AB}}}{{\dot{\varepsilon }}_{0}}\right)\left[1-{\left(\left(T_{\mathrm{AB}}-T_{r}\right)/\left(T_{m}-T_{r}\right)\right)}^{m}\right]$$

where *A*, *B*, *n*, *C*, and *m* are the parameters of the JC constitutive equation; *T*_r_ denotes room temperature (293 K); *T*_m_ stands for the melting temperature (993 K); $\varepsilon_{\mathrm{AB}}$ and ${\dot{\varepsilon }}_{\mathrm{AB}}$ are the equivalent effect variation and equivalent effect variation rate using the von Mises yield criterion equivalent transformation, respectively; and *T*_AB_ represents the average temperature in the shear zone [30].

(8) $$\Phi =\arctan\left(\frac{t_{1}\cos\alpha }{t_{2}+t_{1}\sin\alpha }\right)$$

where *t*_1_ corresponds to the actual turning feed f, α refers to the rake angle of the tool, and *t*_2_ stands for the chip thickness.

(9) $$\varepsilon_{\mathrm{AB}}=\frac{\cos\alpha }{2\sqrt{3}\sin\Phi \cos(\Phi +\alpha )}$$

(10) $${\dot{\varepsilon }}_{\mathrm{AB}}=\frac{C_{0}V\cos\alpha \sin\Phi }{\sqrt{3}t_{1}\cos(\Phi +\alpha )}$$

where $C_{0}$ stands for the ratio of the length of the shear surface to the thickness of the shear zone (which is related to the processing shape and workpiece material, with typical values ranging from 2 to 10 [33]), and *V* represents the cutting speed.

(11) $$C_{0}=\frac{1+2(\pi /4-\Phi )-\tan\theta_{0}}{n_{eq}}$$

(12) $$n_{eq}=\frac{nB{\varepsilon_{AB}}^{n}}{\left(A+B{\varepsilon_{AB}}^{n}\right)}$$

where $\theta_{0}$ denotes the angle between the chip formation force R and shear plane, calculated according to the geometric relationship of the 2D chip forming force in Ref. [34] ($\theta_{0}=\lambda +\alpha +\Phi$), and neq stands for the hardening index.

(13) $$T_{\mathrm{AB}}=T_{r}+\eta \frac{(1-\beta )(F_{z}\cos\Phi -F_{y}\sin\Phi )V\cos\alpha }{\rho Vt_{1}wC_{p}\cos(\Phi +\alpha )}$$

where η(0<η<1) denotes the ratio of the plastic work at the shear surface to the total plastic work, which is taken as 0.9 in this study; w indicates the cutting width, which is equivalent to the actual cutting depth; $C_{p}$ stands for the specific heat capacity; $H_{T}$ refers to the dimensionless thermal coefficient; and β(0<β<1) is the heat distribution coefficient of the shear zone, which can be obtained from the Boothroyd empirical formula [35].

The tangential (*F*_c1_) and normal components (*F*_t1_) of the chip formation force R were calculated using the above parameters [36].

(14) $$\left\{\begin{array}{l} F_{c1}=\frac{\sigma_{\mathrm{AB}}t_{1}w\cos\left(\lambda +\alpha \right)}{\sqrt{3}\sin\Phi \cos\theta_{0}}\\ F_{t1}=\frac{\sigma_{\mathrm{AB}}t_{1}w\sin\left(\lambda +\alpha \right)}{\sqrt{3}\sin\Phi \cos\theta_{0}} \end{array}\right.$$

However, in the actual cutting state of the negative chamfered tool, the actual tool rake angle was 31°, and the cutting force components were obtained through coordinate system transformation and vector decomposition [37]. According to the conversion of cutting force F in xyz and lmn coordinate systems in Ref. [37], the XYZ coordinate system is rotated around the X-axis by the cutting edge inclination angle ($\lambda_{s}$) to obtain a new coordinate system. This new coordinate system is then rotated around the Y-axis by the actual rake angle to obtain the LMN coordinate system, which can be converted to obtain another set of cutting forces acting on the negative chamfer. In the LMN coordinate system, the tangential force (*F_l_*) acts perpendicular to the workpiece on the negative chamfered surface, the normal force (*F_m_*) acts perpendicular to the negative chamfered surface, and the frictional force (*F_n_*) acts parallel to the workpiece on the negative chamfered surface. The friction angle (λ) is calculated using Equation (16) [37].

(15) $$\left\{\begin{array}{l} F_{n}=F_{x}\cos\alpha_{1}-F_{z}\sin\alpha_{1}\\ F_{l}=-F_{x}\sin\lambda_{s}\sin\alpha_{1}+F_{y}\cos\lambda_{s}-F_{z}\sin\lambda_{s}\cos\alpha_{1}\\ F_{m}=F_{x}\cos\lambda_{s}\sin\alpha_{1}+F_{y}\sin\lambda_{s}+F_{z}\cos\lambda_{s}\cos\alpha_{1} \end{array}\right.$$

(16) $$\lambda =\arctan\left(\frac{F_{n}}{F_{m}}\right)$$

(2) Mechanical analysis of chamfered deformation zone

Amorphous alloys exhibit plasticity within a specific temperature range, and the size effects on their mechanical behaviours have been widely studied. The slip-line field model is used to describe the plastic behaviour of materials. Its basic idea is to treat dislocation lines in materials as slip lines and describe the plastic behaviour by simulating the motion and interaction of slip lines in crystals. In the cutting of amorphous alloys, the slip-line field model can be used to explain the deformation and failure of materials, thereby predicting key performance indicators such as cutting force and surface quality. Therefore, it is feasible to apply a slip-line field model to amorphous alloy material properties. Assuming that the metal dead zone in the negative chamfered region forms an angle $\Phi_{cf}$ of the same magnitude as the main shear angle, during the cutting process of negative chamfered tools, owing to the effect of cutting force, the workpiece material generates the main shear deformation zone (AB) and chamfered deformation zone (OH) at the front of the tool. A sector-shaped region (BCO) connects the two zones [38]. Before entering the chamfered deformation zone, the thickness of the cutting layer material is h0. After plastic deformation, its thickness decreases to hf, similar to that observed in the previous tool-face extrusion process, and the force analysis is shown in Ref. [39].

Based on the geometry of the slip-line field in the chamfered edge region, the average plastic strain in the chamfer and the chamfer strain rate [39] are defined as follows:

(17) $$\varepsilon_{cf}=\frac{\tan\Phi_{cf}}{1+\tan\Phi_{\mathrm{cf}}}$$

(18) $${\dot{\varepsilon }}_{cf}=\frac{\varepsilon_{cf}V}{b_{cf}\sin\alpha_{1}}$$

where *b*_cf_ represents the width of the negative chamfer and $\alpha_{1}$ refers to the rake angle of the negative chamfered tool.

Similar to the extrusion process, the ‘metal dead zone’ below the chamfered edge serves as an extension of the cutting tool during the cutting process. The energy dissipated in the chamfered edge region (*E*_cf_) consists of the plastic work (*E*_i_) for extruding the material from thickness ho to hf and the frictional energy (*E*_f_) consumed at the dead metal interface (BO). The temperature Tcf was corrected for the negative chamfered region to derive the corrected flow stress ($\sigma_{cf\text{-}new}$).

(19) $$T_{cf}=T_{AB}+\frac{E_{cf}}{\rho C_{p}}$$

(20) $$E_{cf}=E_{i}+E_{f}=\sigma_{cf}\varepsilon_{cf}+\frac{\cos(2\Phi_{cf})\sigma_{\mathrm{cf}}}{\sqrt{3}\cos^{2}\Phi_{cf}(1+\tan\Phi_{cf})}$$

(21) $$\sigma_{cf\text{-}new}=\left(A+B{\varepsilon_{cf}}^{n}\right)\left(1+C\ln\frac{{\dot{\varepsilon }}_{\mathrm{cf}}}{{\dot{\varepsilon }}_{0}}\right)\left[1-{\left(\left(T_{cf}-T_{r}\right)/\left(T_{m}-T_{r}\right)\right)}^{m}\right]$$

To obtain the flow stress $\sigma_{cf}$ in the negative chamfered zone, a general expression for the material flow stress $\sigma_{1}$ was introduced. $\sigma_{1}$ was statistically processed and obtained from the evaluation of flow stress and strain index values in cutting experiments as a function of the main deformation zone strain and strain rate [39].

(22) $$\sigma_{cf}=\sigma_{1}{\varepsilon_{\mathrm{cf}}}^{n}$$

(23) $$\sigma_{1}=\frac{\sigma_{\mathrm{AB}}}{{\varepsilon_{\mathrm{AB}}}^{n}}$$

The tangential component *F*_cf-c_ and normal component *F*_cf-t_ resulting from the distribution at the metal interface along the dead zone in the negative chamfered region (OH) were calculated using the above parameters [40].

(24) $$\left\{\begin{array}{l} F_{cf\text{-}c}=\left(\tau_{BO\text{-}new}\cos\Phi_{cf}+\sigma_{BO\text{-}new}\sin\Phi_{cf}\right)\frac{b_{cf}\sin\alpha_{1}w}{\cos\Phi_{cf}}\\ F_{cf\text{-}t}=\left(\sigma_{BO\text{-}new}\cos\Phi_{cf}-\tau_{BO\text{-}new}\sin\Phi_{cf}\right)\frac{b_{cf}\sin\alpha_{1}w}{\cos\Phi_{cf}} \end{array}\right.$$

where $\sigma_{CO\text{-}new}$, $\sigma_{BO\text{-}new}$, and $\tau_{BO\text{-}new}$ can be obtained using the Hencky equation and Mohr circle [41].

(25) $$\sigma_{CO\text{-}new}=-K_{\mathrm{cf}\text{-}new}(1+\frac{3\pi }{2})$$

(26) $$K_{\mathrm{cf}\text{-}new}=\frac{\sigma_{\mathrm{cf}\text{-}new}}{\sqrt{3}}$$

(27) $$\sigma_{BO\text{-}new}=-(\sigma_{CO\text{-}new}+K_{cf\text{-}new}\sin(2\Phi_{\mathrm{cf}}))$$

(28) $$\tau_{BO\text{-}new}=\cos(2\Phi_{\mathrm{cf}})K_{\mathrm{cf}\text{-}new}$$

where $\sigma_{CO\text{-}new}$ refers to the flow stress in the CO region, $\sigma_{\mathrm{cf}\text{-}new}$ stands for the flow stress in the chamfered edge region, $\sigma_{BO\text{-}new}$ indicates the flow stress in the BO region, and $\tau_{BO\text{-}new}$ denotes the shear stress in the BO region.

(3) PSO for solving JC constitutive parameters

The PSO algorithm was first proposed by Kennedy and Eberhart [42]. Each particle has two attributes, namely the position and velocity of the vector, which represent the direction and magnitude of the velocity. In multidimensional search spaces, the position and velocity are also multidimensional vectors. In this problem, *A*, *B*, *n*, *C*, and *m* in the JC constitutive equation are five parameters representing five-dimensional spaces. If the position of each parameter is represented by *X_i_* and the speed by *V_i_*, then

(29) $$N_{i}=\left[X_{i},V_{i}\right]$$

(30) $$\left\{\begin{array}{l} X_{i}=\left[Ax_{i},Bx_{i},nx_{i},Cx_{i},mx_{i}\right]\\ V_{i}=\left[Av_{i},Bv_{i},nv_{i},Cv_{i},mv_{i}\right] \end{array}\right.$$

An individual with the above attributes is called particle i. The collection of j individuals forms the entire population, denoted as *P*.

(31) $$P=\left[N_{1},N_{2},N_{3}\cdots N_{j}\right]$$

By substituting the position as a variable into the objective function, a value that can be used to evaluate the results can be obtained, as expressed in Equation (32).

(32) $$f(X_{i})=f(Ax_{i},Bx_{i},nx_{i},Cx_{i},mx_{i})$$

An initial population was created for the optimisation process, with each particle randomly assigned a value within the boundary constraint variables (*a_i_*, *b_i_*) for the following randomly assigned initial position and velocity. The boundary range for this problem was set based on the parameters of the JC constitutive equation derived in Section 2.2.1. The current position of particle i was first recorded as *X_i_*(*t*), and its new velocity *V_i_*(*t* + 1) was calculated based on its current velocity *V_i_*(*t*). After the corresponding velocity each particle was updated, its position *X_i_*(*t* + 1) was updated using the following formulas [43]:

(33) $$X_{i}(t=0)=a_{i}+rand_{i}(b_{i}-a_{i})$$

(34) $$V_{i}(t+1)=c_{1}V_{i}(t)+c_{2}rand_{1}(X_{gbest}-X_{i}(t))+c_{3}rand_{2}(X_{zbest}-X_{i}(t))$$

(35) $$X_{i}(t+1)=X_{i}(t)+V_{i}(t+1)$$

where *c*_1_ represents the inertia weight, *c*_2_ refers to the self-learning factor, *c*_3_ stands for the group learning factor, and Rand is randomly chosen between 0 and 1.

During the generation of the particle population, the best position *X*_gbest_ of the current population and the historical best fitness value *X*_zbest_ were updated.

**Figure 2:**
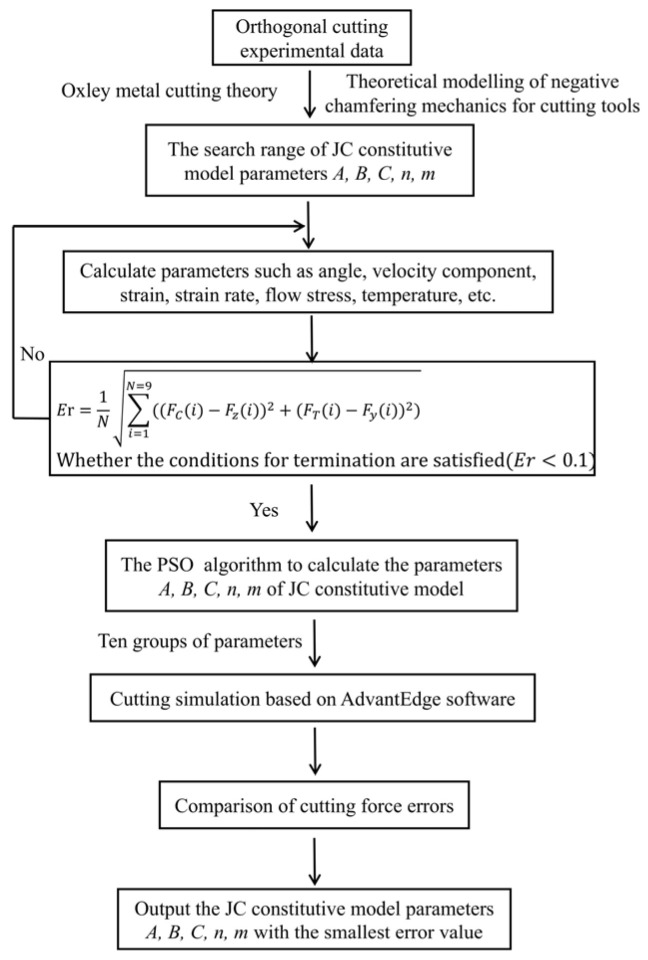
Derivation and optimisation of JC constitutive model parameters.

In this optimisation problem, nine sets of cutting conditions and test results were known, and variables such as shear angle, friction angle, equivalent variation, mean temperature in the shear zone, and flow stress were calculated using equations. These variables were represented by multiple sets of equations containing the parameters (*A*, *B*, *n*, *C*, and *m*) to be solved. By substituting these equations into the cutting force formula, the theoretical cutting force containing the desired parameters and the force generated by the negative chamfered deformation zone could be calculated. To minimise the error between experimental and theoretical cutting forces, the objective function and corresponding constraint functions must be defined. The root mean square of the error between the experimental and theoretical cutting forces was calculated as an objective function, as expressed in Equation (36), and the constraint function was expressed as shown in Equation (37). The problem of solving for the parameters to be determined was converted into a parametric optimisation problem, and the specific solution process is illustrated in Figure 2.

Objective function:

(36) $$Er=\frac{1}{N}\sqrt{\sum_{i=1}^{N=9}\left({\left(F_{C}(i)-F_{z}(i)\right)}^{2}+{\left(F_{T}(i)-F_{y}(i)\right)}^{2}\right)}$$

Constraint function:

(37) $$2\leq C_{0}\leq 10$$

A program was written in MATLAB (R2020a) based on the objective function and constraint conditions of this problem. According to the process depicted in Figure 2, the cutting parameters for each group, experimental data from Table 1, Vit1 thermophysical parameters from Table 1, and search ranges for the JC constitutive parameters in Table 2 were used as input. The PSO algorithm was used to optimise the objective function, and a JC constitutive equation suitable for actual cutting was obtained. The parameter settings for the PSO algorithm are listed in Table 3. According to the PSO algorithm optimisation solution iteration, the optimisation results were not unique. The program was run ten times to obtain ten sets of JC parameters, and the errors were compared separately.

## 3. Finite Element Modelling

### 3.1. Pre-Processing Modelling

**Figure 3:**
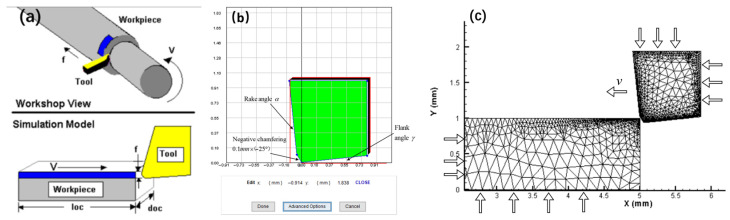
FEM model. (**a**) Cutting simulation model; (**b**) geometric tool; (**c**) meshing.

A finite element analysis model was established using AdvantEdge software (v7.1-002), as displayed in Figure 3a. In this program, the cutting length is denoted as *loc*, and the cutting depth is represented by *f*, which is the actual feed rate during cutting. The cutting width is expressed as doc, which is the actual cutting depth during the cutting process, and the cutting speed is expressed as *v*_c_ (m/min). The built-in tool geometry editor constructed a negative chamfered structure of the tool with fixed constraints applied to the upper and right ends, as shown in Figure 3b. CBN was used as the cutting tool material.

The M-C friction model of the FEM was adopted. Incorporating the experimental data of Vit1 orthogonal cutting into the theoretical calculation formula for the negative chamfered friction angle revealed that the friction coefficients obtained were all above 1, contradicting the conventional understanding that the theoretical friction coefficient is less than 1. Different friction coefficients were set using the software, and it was verified that a friction coefficient of 0.1 yielded cutting forces closer to actual values and chip characteristics more similar to real-world observations. Therefore, the coefficient of friction was set to 0.1 in subsequent simulations.

Both geometric and physical criteria define the chip separation process in cutting simulations, significantly influencing the accuracy of the cutting model. Geometric guidelines stipulate that the tip-to-tip unit node should be determined according to a critical value. When the distance is less than this specified critical value, the node separates from the workpiece substrate, producing a chip morphology [44]. The physical criterion uses the relevant physical quantities of the material to determine whether the node element is separated from the workpiece substrate. By comparing these physical quantities to the limits that the material can withstand, it is possible to determine if the node has reached a state of damage. The commonly used physical criteria include stress fracture, equivalent plasticity, and Rice and Tracer three-dimensional stress criteria [45]. Compared with the geometric separation criterion, the physical criterion not only reflects the mechanical properties of the machined material but also accurately represents its physical properties, making simulation machining more aligned with actual machining conditions. AdvantEdge stores all strain information as tensile strain data by default. In this study, the cutting model for the Vit1 amorphous alloy was developed using the three-dimensional stress criterion from the physical separation criterion, which is given by Equation (38).

(38) $$\int_{0}^{{\bar{\varepsilon }}_{f}}\exp(1.5\frac{\sigma_{m}}{\bar{\sigma }})d\bar{\varepsilon }=C_{1}$$

where ${\bar{\varepsilon }}_{f}$ indicates the strain at which failure occurs under the current stress state, $\frac{\sigma_{m}}{\bar{\sigma }}$ represents the parameter of the three-dimensional stress magnitude, and $C_{1}$ refers to the critical failure value.

When performing a simulation analysis, the workpiece must first be meshed. In this simulation, the mesh size for the workpiece and tool ranged from 0.001 to 0.01 mm, with a mesh grading of 0.4, as depicted in Figure 3c. Boundary constraints were set at the bottom and left sides of the workpiece. A given tool was set with a speed along the x-negative direction to enable it to cut the workpiece. To better simulate the actual machining process, the initial temperature of both the workpiece and tool was set to 20 °C.

### 3.2. Construction of Actual JC Constitutive Model Parameters

The resulting ten sets of JC parameters were entered into the AdvantEdge simulation software as a custom material to conduct a simulation study of the two-dimensional cutting process. The simulation results were subsequently used to compare and analyse the experimental data. In the post-processing phase, the cutting forces were extracted from the cutting stability zone and averaged to obtain the final simulation result. The average error values of the cutting force data obtained from the simulation for three sets of experimental data (Nos. 1, 2, and 3 in Table 1) are presented in Table 4. The JC constitutive model parameters with the minimum cutting force errors were identified.

It was found that the solved A values were all smaller than those calculated from the stress–strain curves. This is because Vit1 cutting is a heating process, and an increase in temperature reduces the yield strength. The small m ratio indicates that Vit1 has a high sensitivity to plastic strain and temperature. The JC constitutive model simulated for No. 4 in Table 4 had the smallest average error of force simulation, which was 10.81%. By averaging the aforementioned parameters, an average error of 17.34% was obtained for the force simulated using the JC constitutive model. Therefore, using the JC constitutive model parameters of No. 4 in Table 4, the final JC model equation for Vit1 was obtained, as shown in Equation (39). This equation is more consistent with the finite element analysis of the cutting process than the data fitted under the stress–strain curve and can greatly improve the simulation accuracy.

(39) $$\sigma =\left(850.1+519.5\varepsilon^{0.2131}\right)\left(1+0.1061\ln\frac{\dot{\varepsilon }}{{\dot{\varepsilon }}_{0}}\right)\left[1-{\left(\frac{T-T_{r}}{T_{m}-T_{r}}\right)}^{0.0813}\right]$$

## 4. Results and Discussion

### 4.1. Comparison of Cutting Forces

**Figure 4:**
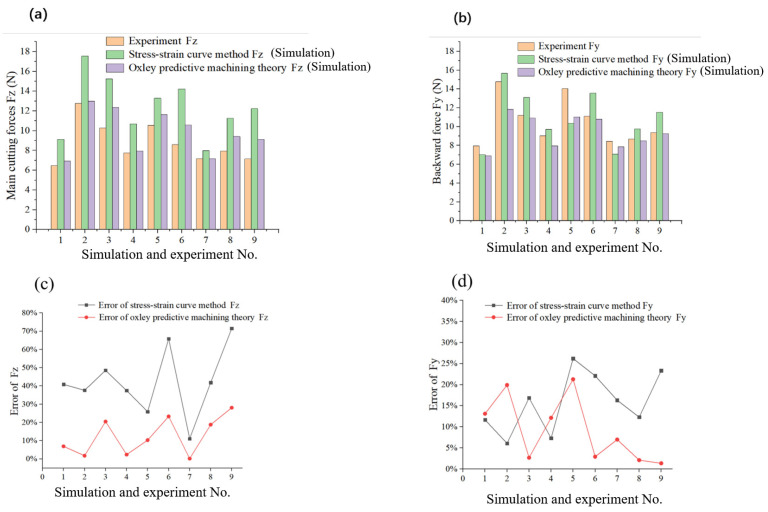
Comparison of the experimental and simulation results of cutting force. (**a**) Fz obtained by experiment and simulation; (**b**) Fy obtained by experiment and simulation; (**c**) Error of Fz obtained by experiment and simulation; (**d**) Error of Fy obtained by experiment and simulation.

To validate the applicability of the JC constitutive equations obtained through PSO algorithm optimisation in actual Vit1 cutting operations, the parameters of the JC constitutive equation derived from fitting the stress–strain curve and those obtained through the PSO algorithm calculation were input into the simulation software. To ensure simulation reliability, the tool parameters and cutting conditions were kept constant. Based on the experimental parameters listed in Table 1. The results of the simulated cutting force obtained by JC constitutive model through the stress–strain method and Oxley predictive machining theory derivation method were compared with the experimental results and visualised in error plots, as shown in Figure 4.

In Figure 4, it can be observed that the main cutting forces obtained from the simulation are slightly greater than the actual values, which may be due to differences in chip separation criteria. The JC constitutive and damage equations are commonly used together to accurately and reliably describe changes in material constitutive and damage properties during the cutting process in finite element simulations. However, this study only employs the JC constitutive model, whereas the AdvantEdge software uses a three-dimensional stress criterion for chip separation, potentially introducing errors. Furthermore, the cutting simulation employs a fixed Coulomb friction model. Owing to the dynamic nature of the cutting process and the potential for the friction coefficient at the tool–chip interface to change because of various factors, the friction coefficient at the tool–chip interface is not a constant value. Additionally, the simulation did not account for the grain content in the CBN tool or the type of bonding agent, which might have contributed to the slight differences between the simulated and actual main cutting forces.

Comparing the force results in Figure 4a,b, the simulation results obtained using the Oxley predictive machining theory to derive the JC constitutive equation parameters are more accurate than those using the stress–strain curve method. The simulated cutting forces align better with the experimental results. The errors in Fz and Fy are shown in Figure 4c,d. According to the comparative error analysis, the main cutting and backward forces obtained by Oxley’s method to extrapolate the parameters of the JC constitutive equation yield smaller errors compared with the stress–strain curve method, with average errors of 12.461% and 9.161%, respectively. In contrast, the average errors of the main cutting and backward forces obtained using the stress–strain curve method for fitting the JC constitutive equation parameters are 42.305% and 15.789%, respectively. These results demonstrate that the main cutting and backward forces in the actual cutting process can be simulated more accurate using Oxley’s method to derive the JC constitutive equation parameters, which offers higher reliability and is more suitable for the actual cutting process.

### 4.2. Verification of Chip Morphology in FEMs

In the process of machining brittle materials, granular and crushing chips are usually generated. When machining plastic materials, continuous ribbon and serrated chips are common chip morphologies [46]. Ribbon chips are considered ideal and stable cutting products. In contrast, serrated chips originate from mechanical instability during the cutting process and are characterised by periodic fluctuations in cutting force and temperature.

**Figure 5:**
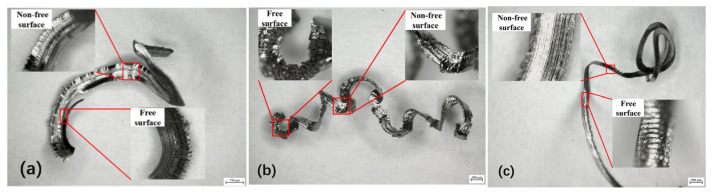
Chip morphology under different cutting conditions (*a*_p_ = 0.2 mm). (**a**) *v*_c_ =10 m/min, *f* = 0.02 mm/r; (**b**) *v*_c_ = 10 m/min, *f* = 0.06 mm/r; (**c**) *v*_c_ = 20 m/min, *f* = 0.06 mm/r.

The chip morphology obtained under different cutting conditions was observed using a German Leica ultra-depth-of-field 3D microscope, as illustrated in Figure 5. The free surface of the chips obtained under low feed rates presents a layered scale-like shape, while the non-free surface is smooth and delicate. This is attributed to the sensitivity of amorphous alloys to temperature and stress. Although Vit1 exhibits brittleness at room temperature, an increase in temperature during the cutting process can cause it to display a certain degree of plasticity. As the tool feed movement increases, the extrusion force acting on the cutting layer of Vit1 by the tool gradually increases, resulting in some elastic deformation in the cutting layer material.

The cutting layer material fractures when the extrusion force of the cutting tool exceeds the yield strength limit of the Vit1 material. The material of the cutting layer undergoes plastic sliding under the thrust of the cutting tool and is then pushed away from the workpiece machining surface. The extrusion pressure acting on the cutting layer material is instantly released. When the cutting layer is very thin, some of the material is pushed away by the tool without plastic deformation, which then flows towards the rake face to generate fish scale-shaped chips. Figure 5b,c shows that the free surface of the chips exhibits a scale shape. As the actual thickness of the cutting layer increases owing to the increased feed rate, the spacing between chip scales also increases. In addition, the non-free surface of the chips becomes slightly rougher. Based on these observations, it can be preliminarily concluded that the smoothness of the non-free surface is related to the cutting speed and feed rate. The non-free surface flows from the main cutting edge of the tool to the rake face, generating significant frictional heat. At high temperatures, Vit1 undergoes significant plastic deformation, leading to a continuous chip morphology during the expulsion process and a smooth non-free surface. However, increased cutting speeds and feed rates generate heat that can soften chips, causing tangling and curling. Additionally, the softened chips can carry away some of the tool material, resulting in more scratches on the non-free surface.

**Figure 6:**
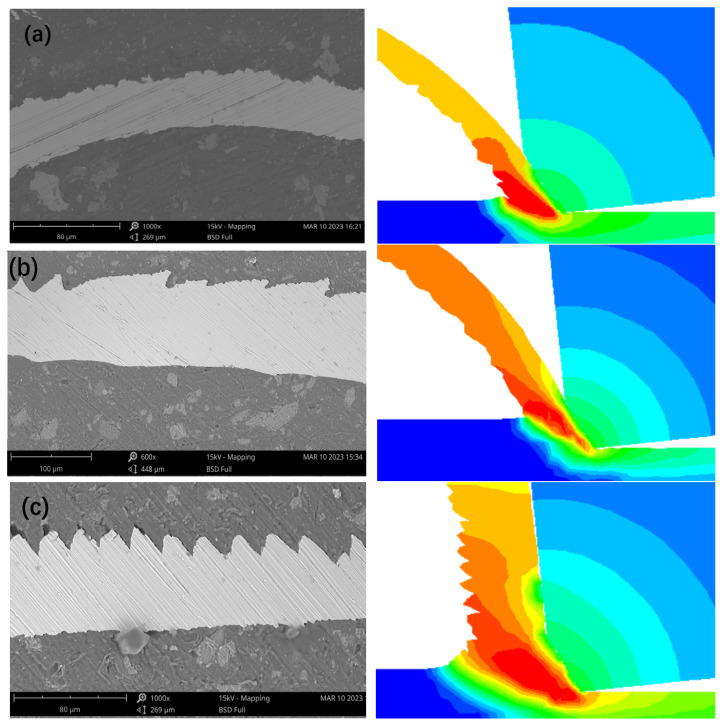
Comparison between experimental and simulated chip morphologies. (**a**) *v*_c_ = 10 m/min, *f* = 0.02 mm/r, *a_p_* = 0.2 mm; (**b**) *v*_c_ = 10 m/min, *f* = 0.06 mm/r, *a_p_* = 0.2 mm; (**c**) *v*_c_ = 20 m/min, *f* = 0.06 mm/r, *a_p_* = 0.2 mm.

Based on the comparison results of simulated and experimental cutting forces in the above Section 4.1, the proposed constitutive model that uses Oxley’s method to derive the JC constitutive equation parameters was compared and verified further using the simulation and experimental results of the chip morphology, as shown in Figure 6.

In Figure 6a,b, it can be observed that the chip morphologies obtained from both the experiments and finite element simulations are very similar and show lamellar ribbons, and the free surfaces exhibit a certain amount of concavity and convexity. The lamellar cutting interval in Figure 6b is slightly larger than that in Figure 6a and has a somewhat serrated shape. Figure 6c shows that the simulated and experimental chips evolve into smaller serrated shapes at higher cutting speeds, which further demonstrates the reliability of the obtained JC constitutive model parameters. The established finite element simulation model is reasonable and can be applied to the analysis of the Vit1 cutting process and chip formation mechanism. The chip morphology simulation reveals that at lower cutting speeds, chips are pushed out along the negative chamfer of the tool, which facilitates chip removal from the machined surface. However, at higher cutting speeds, the chips flow over the negative chamfer and discharge along the rake face. This suggests a rougher non-free surface of the chip at higher cutting speeds. Additionally, a metal dead zone exists before the negative chamfer of the tool, effectively squeezing the chips.

### 4.3. Mechanism of Chip Formation 

**Figure 7:**
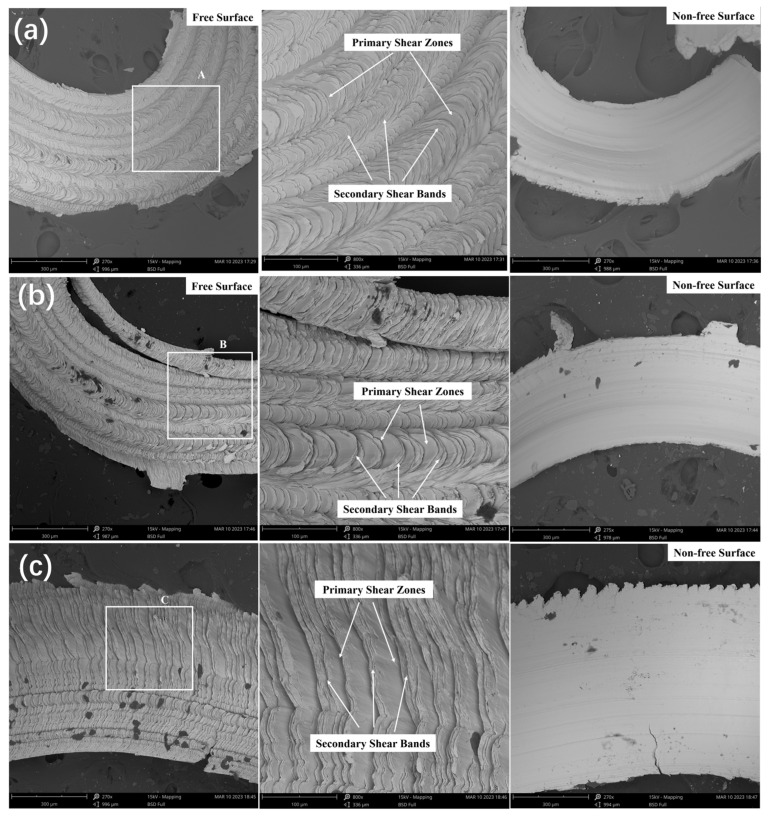
SEM images of free and non-free surfaces of chips. (**a**) *v*_c_ = 10 m/min, *f* = 0.02 mm/r, *a_p_* = 0.2 mm; (**b**) *v*_c_ = 10 m/min, *f* = 0.06 mm/r, *a_p_* = 0.2 mm; (**c**) *v*_c_ = 20 m/min, *f* = 0.06 mm/r, *a_p_* = 0.2 mm. (The second subgraph of Figure 7a, Figure 7b, and Figure 7c are amplification of box A, B, and C, respectively).

The aforementioned analysis indicates that the chips formed from Vit1 at low speeds are primarily lamellar and gradually evolve into serrated chips as the speed increases. To further analyse the chip formation mechanism, the chips produced under the aforementioned cutting conditions were observed using SEM, and the free and non-free surface morphologies under different cutting conditions were obtained, as shown in Figure 7.

The main vein patterns of the chips are separated under different feed conditions, as illustrated in Figure 7a,b. This is because of the low cutting speed, which causes slower extrusion and severe material accumulation, resulting in the formation of such veins. As the feed rate increases, the chips are easily torn along the main vein. In contrast, a lower feed rate results in more delicate, fish-scale-shaped chips. In Figure 7a, it is impossible to distinguish between the primary and secondary shear bands, which indicates a ribbon-like chip morphology. From Figure 7b, it can be observed that the distance between the fish-scale-like slices increases, and the primary and secondary shear bands are already well distinguished, indicating that the chip morphology tends to change towards a serrated shape. In Figure 7c, the fish-scale-like slices disappear, presenting an overall thin slice-like primary shear band. These fractures form by the direct plastic deformation expansion between the cutting layer material and uncut layer material, which is the chip serrated unit in Figure 6. After fracture, the cutting layer material is squeezed and pushed away from the workpiece by the tool. A portion of the material being pushed away undergoes a small displacement relative to the microcracks, forming and stacking many small folds on the cracks, called secondary shear bands.

The displacement extension of the secondary shear bands is irregular, but the final sliding direction is along the primary shear band. No molten droplets are observed in the free surface gaps of the chips under the aforementioned cutting conditions, indicating that the generated cutting heat does not cause material melting. The non-free surface of the chip in Figure 7a is smooth and neat, whereas that in Figure 7b,c has black spots (which may be material shed by the tool) and side burrs. At higher cutting speeds, the chips exhibit more lateral tearing burrs and significant chip edge cracking.

**Figure 8:**
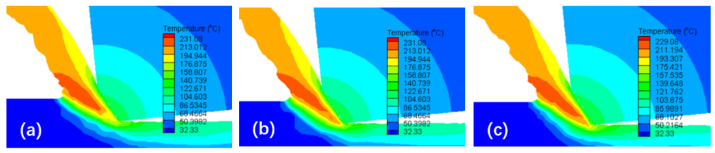
Simulated temperature distribution during chip formation. (**a**) *t*_1_ = 0.0053 s; (**b**) *t*_2_ = 0.0056 s; (**c**) *t*_3_ = 0.0059 s.

Figure 8 displays the simulated temperature distribution during chip formation under the conditions of *v*_c_ = 10 m/min, *f* = 0.06 mm/r, and *a*_p_ = 0.2 mm. It can be observed that in the initial stage of the cutting process, the temperature of the material at the negative chamfer of the tool starts to increase, exceeding the temperature of other uncut layer materials and extending to the free surface of the chip. As the tool moves towards the workpiece, the material of the cutting layer slides away from the adiabatic shear bands and is pushed away from the workpiece, forming chips and creating a new cutting layer in the contact area between the tool and workpiece. The aforementioned temperature change process is repeated, and the existence of adiabatic shear bands becomes evident.

**Figure 9:**
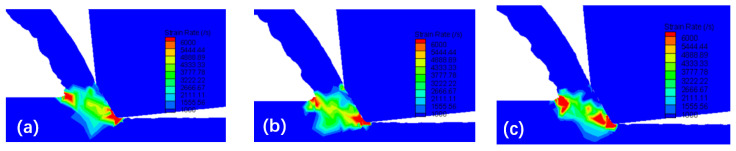
Simulated strain rate distribution during chip formation. (**a**) *t*_1_ = 0.0053 s; (**b**) *t*_2_ = 0.0056 s; (**c**) *t*_3_ = 0.0059 s.

Figure 9 illustrates the variation in strain rate at each stage of the chip formation process, which clearly shows the generation of lamellar chips. The maximum strain rate is concentrated in the shear transition zone, extending from the main cutting edge of the negative chamfer of the tool to the free surface of the chip; however, the strain rate is not uniformly distributed. The middle part of the shear transition zone is relatively small, indicating that a portion of the material is extruded and that the highest strain rate is located below the adiabatic shear bands. This is because only when the material strain rate in the shear transition zone exceeds a certain critical value does the cutting layer form an adiabatic shear zone, subject to high temperatures and stresses before shear slip can occur.

**Figure 10:**
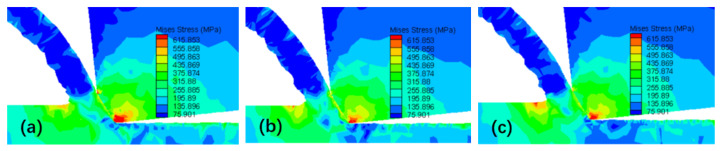
Simulated stress distribution during chip formation. (**a**) *t*_1_ = 0.0053 s; (**b**) *t*_2_ = 0.0056 s; (**c**) *t*_3_ = 0.0059 s.

Figure 10 shows the change in stress during the simulated chip formation process. The chip stresses at the negative chamfer of the tool and the contact zone with the chip are relatively high. During the process of chip detachment from the rake face, the chip is subjected to frictional force and is continuously pushed forward by the newly formed cutting layer material towards the cutting edge. Consequently, the stress of the material in the cutting deformation zone is relatively high. During the cutting process, a portion of the uncut material in front of the chips experiences significant stress. The negative chamfered structure of the tool results in a larger actual rake angle, which facilitates chip evacuation but squeezes the unmachined surface.

**Figure 11:**
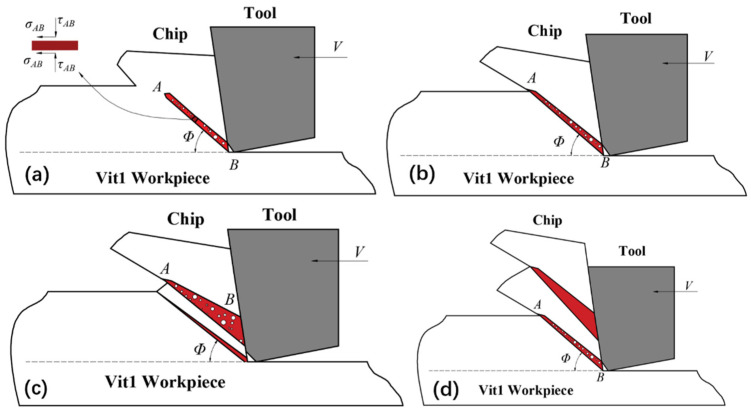
Simplified model of the chip formation process. (**a**) First stage; (**b**) second stage; (**c**) third stage; (**d**) fourth stage.

According to the above analysis of the material strain rate, temperature, and stress in the shear deformation zone, the process of transforming lamellar chips into serrated chips is induced by the coupling of pressure and temperature, which is essentially the process of forming regular shear bands within the adiabatic shear zone. Based on the free volume theory, the Vit1 chip production process was separated into three stages, as depicted in Figure 11.

First stage: When an external force acts on the atomic cluster in Vit1, a stress region is formed around the cluster, disrupting its stability. The increase in free volume concentration and temperature in these stress regions also promotes the diffusion of the free volume, as represented by the white circle in Figure 11. As the free volume accumulates and breaks, these atomic clusters form high-stress regions, which are localised adiabatic shear bands generated inside the amorphous alloy. The first lamellar unit is also produced concurrently, as illustrated in Figure 11a.

Second stage: The temperature, strain rate, and stress of the material in the shear deformation area increase drastically as a result of the continuous cutting and extrusion of the cutting tool. The cutting stresses generated by the applied load either compress or stretch the atomic clusters in the region, and an increase in temperature causes the material to deform plastically. As shown in Figure 11b, the adiabatic shear bands extend from the negative chamfered contact zone to the free surface of the chip along the reverse direction of the principal stress. Under the influence of adiabatic shear, the material bulges and forms lamellar chip units, morphologically representing the plastic deformation and shear slip experienced by the material in the constrained area to generate chips.

Third stage: As the tool advances, additional material enters the shear deformation zone after the first laminated chip unit is generated. Some materials are extruded to form secondary shear bands before plastic deformation occurs. These secondary shear bands compress and push up the second layered chip unit, causing it to slide upwards along the negative chamfer and rake face. Consequently, a second shear surface is created, and the subsequent lamellar chip unit begins to form. This process is continuously repeated, culminating in the formation of the entire lamellar chip, as illustrated in Figure 11c,d.

## 5. Conclusions

Based on orthogonal cutting experiments and negative chamfer mechanical modelling, the JC constitutive model parameters of Vit1 material were determined through calculations, and conclusions can be drawn as follows:

(1) By establishing a finite element simulation model for the cutting of Vit1 material and comparing it with the cutting experimental results, the chip morphology of FEM was found to be consistent with that of the experimental results. The average errors of Fz and Fy obtained by fitting the parameters of the JC constitutive equation with the Oxley predictive machining theory were 12.461% and 9.161%, respectively. In contrast, the average errors of Fz and Fy obtained by fitting the parameters of the JC constitutive equation with the stress–strain curve method were 42.305% and 15.789%, respectively.
(2) Based on the observation of the micromorphology of the chips using SEM, at low cutting speeds, the serration shape of the chips was not clear, showing a continuous lamellar strip. However, at high cutting speeds, when cutting Vit1, obvious serrated chip characteristics were observed, with a relatively rough non-free surface.
(3) The transformation of lamellar chips into serrated chips resulted from a combination of adiabatic shear, shear slip, and plastic deformation. Below the primary shear zone, several secondary shear zones existed where shear displacement did not occur. These secondary shear zones were formed by extrusion. Based on the free volume theory, the formation of Vit1 chips was divided into three stages, and the mechanism of chip deformation was revealed while cutting Vit1.

## Figures and Tables

**Table 1:** Orthogonal experimental cutting parameters.

No.	Cutting Speed *v*_c_ (m/min)	Feed Rate *f* (mm/r)	Actual Cutting Depth Δ*a_p_* (mm)	Chip Thickness *t*_2_ (mm)
1	10	0.02	0.170	0.0508
2	10	0.04	0.185	0.0939
3	10	0.06	0.170	0.1306
4	15	0.02	0.175	0.0551
5	15	0.04	0.175	0.0891
6	15	0.06	0.190	0.1256
7	20	0.02	0.180	0.0517
8	20	0.04	0.185	0.0919
9	20	0.06	0.180	0.1189

**Table 2:** Search ranges of five parameters in the JC constitutive equation.

*A* (MPa)	*B* (MPa)	*n*	*C*	*m*
500~2500	450~1500	0.001~1	0.001~2	0.001~2

**Table 3:** Parameter settings for the PSO algorithm.

Number of Initial Populations	Spatial Dimension	Maximum Number of Iterations	Speed Limit	Inertia Weight *c*_1_	Self-Learning Factor *c*_2_	Group Learning Factor *c*_3_	Deviation of Fitness Value
100	5	300	[−1, 1]	0.8	0.5	0.5	0.001

**Table 4:** Average error of the JC constitutive model.

No.	*A* (MPa)	*B* (MPa)	*n*	*C*	*m*	Error of No. 1 in Table 1	Error of No. 2 in Table 1	Error of No. 3 in Table 1	Average Error
1	869.1	971.8	0.8981	0.0204	0.0341	0.3021	0.2615	0.3019	0.2885
2	1073.7	1181.1	0.3796	0.0217	0.0256	0.2908	0.3614	0.1323	0.2615
3	806.2	671.8	0.1061	0.2115	0.0912	0.2498	0.2924	0.3287	0.2903
4	850.1	519.5	0.2131	0.1061	0.0813	0.1001	0.1084	0.1158	0.1081
5	887.9	788.5	0.7460	0.0116	0.0448	0.2170	0.2655	0.1710	0.2179
6	952.8	537.2	0.4465	0.1568	0.0634	0.1450	0.0949	0.1920	0.1439
7	904.8	610.7	0.4944	0.0799	0.0498	0.0345	0.2638	0.1290	0.1424
8	844.2	1122.3	0.7091	0.0901	0.0423	0.2508	0.1997	0.2378	0.2294
9	868.3	776.2	0.5824	0.0343	0.0634	0.1305	0.2049	0.1345	0.1566
10	830.2	591.7	0.7633	0.042	0.0545	0.1441	0.2417	0.1292	0.1717
Average	888.7	777.1	0.5249	0.0774	0.0550	0.1691	0.2088	0.1424	0.1734

## Data Availability

The original contributions presented in this study are included in the article. Further inquiries can be directed to the corresponding author.
